# LC-MS/MS-based serum proteomics reveals a distinctive signature in a rheumatoid arthritis mouse model after treatment with mesenchymal stem cells

**DOI:** 10.1371/journal.pone.0277218

**Published:** 2022-11-04

**Authors:** Namhee Jung, Soyoung Park, TaeHo Kong, Hwanhee Park, Woo Min Seo, Seunghee Lee, Kyung-Sun Kang

**Affiliations:** 1 Stem Cell and Regenerative Bioengineering Institute, Global R&D Center, Kangstem Biotech Co., Ltd., Geumcheon-gu, Seoul, South Korea; 2 Adult Stem Cell Research Center, College of Veterinary Medicine, Seoul National University, Seoul, South Korea; 3 Research Institute for Veterinary Science, College of Veterinary Medicine, Seoul National University, Seoul, South Korea; TotiCell Limited, Bangladesh, BANGLADESH

## Abstract

Mesenchymal stem cells (MSCs) are known to be able to modulate immune responses, possess tissue-protective properties, and exhibit healing capacities with therapeutic potential for various diseases. The ability of MSCs to secrete various cytokines and growth factors provides new insights into autoimmune-diseases such as rheumatoid arthritis (RA). RA is a systemic autoimmune disease that affects the lining of synovial joints, causing stiffness, pain, inflammation, and joint erosion. In recent years, MSCs-based therapies have been widely proposed as promising therapies in the treatment of RA. However, the mechanism involved in disease-specific therapeutic effects of MSCs on RA remains unclear. To clarify the mechanism involved in effects of MSCs on RA, proteomic profiling was performed using an RA mouse model before and after treatment with MSCs. In this study, treatment efficacy of human umbilical cord blood-mesenchymal stem cells (hUCB-MSCs) was confirmed using a type II collagen-induced arthritis (CIA) mouse model. Results of measuring incidence rates of arthritis and clinical arthritis index (CAI) revealed that mice administrated with hUCB-MSCs had a significant reduction in arthritis severity. Proteins that might affect disease progression and therapeutic efficacy of hUCB-MSC were identified through LC-MS/MS analysis using serum samples. In addition, L-1000 analysis was performed for hUCB-MSC culture medium. To analysis data obtained from LC–MS/MS and L-1000, tools such as ExDEGA, MEV, and DAVID GO were used. Results showed that various factors secreted from hUCB-MSCs might play roles in therapeutic effects of MSCs on RA, with platelet activation possibly playing a pivotal role. Results of this study also suggest that SERPINE1 and THBS1 among substances secreted by hUCB-MSC might be key factors that can inhibit platelet activation. This paper is expected to improve our understanding of mechanisms involved in treatment effects of stem cells on rheumatoid arthritis.

## Introduction

Rheumatoid arthritis (RA), a chronic autoimmune inflammatory disorder with an incompletely understood etiology, affects roughly 1% of the world’s population [1]. Although the pathogenesis of RA is not precisely known, its most important features include inflammation of the synovial lining, subsequent abnormal infiltration, and activation of diverse immune cells into the synovial membrane. These features are associated with an imbalance between pro- and anti-inflammatory cells. They are also associated with numerous immune responses and mechanisms involving T cells, B cells, dendritic cells (DC), and fibroblast synovial cells (FLS) [2–4]. Another etiological mechanism of RA is an increase in platelets due to expression of many genes in the initial acute phase response [5–8]. In RA patients, elevated levels of platelet count or platelet indices (mean platelet volume, MPV) have been reported compared to the healthy controls with a positive correlation with DAS28 [7]. Platelet activation is accompanied by the formation of platelet microparticles (MPs), and, similarly, a higher number of MPs has been associated with disease activity [9, 10]. David’s group has demonstrated the *in vivo* evidence for the association with the platelet activation and development of arthritis using the K/BxN serum transfer model of inflammatory arthritis. In addition, they also suggested the joint inflammation mechanism through which platelet MPs expressed membrane-bound IL-1a and IL-1b followed by stimulation of FLSs to release pro-inflammatory cytokines such as IL-6 and IL-8 [11].

Mesenchymal stem cells (MSCs) originating from diverse tissues such as umbilical cord blood, bone marrow, adipose tissue, tonsil, and other tissues play important roles in natural repairing, immune regulation, self-renewal capability, and pluripotency. MSCs have been widely investigated for cell-based therapy. Active research using MSCs is still ongoing [12]. Immune modulation is a key function of MSCs which could alleviate inflammatory responses in damaged organs and tissues [13]. In recent years, MSC-based therapies have become an interesting therapeutic opportunity for the treatment of RA due to their capacity to potently modulate immune responses in both preclinical studies [14] and clinical studies [15]. Possible therapeutic mechanisms involved in the effect of MSCs on RA include cell to cell contact between MSCs and immune cells and induction of death of effector lymphocytes. In addition, MSCs can secrete immune modulators such as transforming growth factor (TGF), indoleamine 2,3-dioxygenase (IDO), and prostaglandin E2 (PGE2). They can also induce regulatory T (Treg) cells or inhibit pro-inflammatory factors including IL-6 and TNF-alpha [16–18].

Liquid chromatography tandem mass spectrometry (LC-MS/MS) can classify proteins in a sample by liquid chromatography and measure amounts of proteins with a tandem mass spectrometer. Nowadays, LC-MS/MS is widely used to identify specific proteins in various pools that are not known yet. It can be used to find new serum biomarkers. This analysis has been used in clinical laboratories and various expanding fields [19]. L-1000, an antibody-based cytokine array, is a tool for detecting and profiling levels of various proteins, including cytokines, growth factors, and chemokines.

Recently, proteome analysis has been used to better understand synovial fluid flowing in RA. However, the underlying mechanism of RA has not been fully understood due to its complexity. Few studies have focused on serum protein difference in RA. In this study, cytokines secreted by hUCB-MSCs were analyzed using L-1000 and protein changes in serum after administering hUCB-MSCs to an RA mouse model were analyzed using LC-MS/MS. The objective of this study was to understand effects of hUCB-MSCs as therapeutic agents on RA with a proteomic approach.

## Materials and methods

### Induction and clinical assessment of collagen-induced arthritis (CIA)

**Fig 1:**
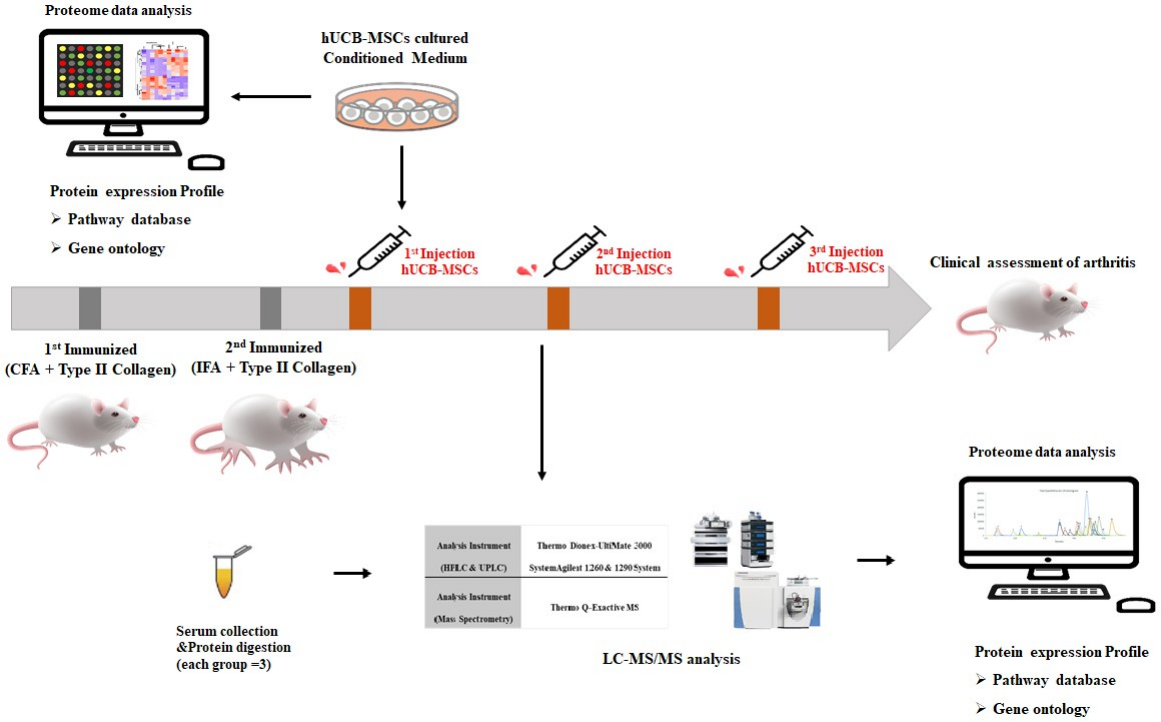
Schematic outline of therapeutic efficacy of hUCB-MSCs on CIA mice model of RA and proteome data analysis by LC–MS/MS and L-1000. Type 2 collagen was injected into mice twice to prepare the CIA mice model (n = 4 for each group). The hUCB-MSCs were injected once a week, for a total of 3 times. For serological analysis (LC-MS/MS analysis), serum was collected by the cardiac blood collection method one week after the first injection of hUCB-MSCs. Clinical assessment was performed until the end of the experiment. In order to analyze factors secreted by hUCB-MSCs, conditioned media of hUCB-MSCs were analyzed by L-1000 array.

All animal care and experimental procedures were approved by Kyungpook National University Institutional Animal Care and Use Committee (Approval Number: KNU 2021–0009). They were conducted following the institutional guideline for animal welfare [20]. Male DBA/1J mice were obtained from SLC (Hamamatsu, Japan) and maintained under specific pathogen free conditions with a controlled environment (temperature of 25°C, 12/12 h light-dark cycle) at the animal facility of Kyungpook National University School of Medicine. All efforts were made to minimize animal suffering. Collagen-induced arthritis (CIA) was induced and scored according to previous reports with minor modifications [21–23]. Briefly, DBA/1J mice between the age of 6 and 8 weeks were immunized intradermally with 100 μg of bovine type II collagen (Chondrex, cat. 20021) in Complete Freund’s adjuvant (CFA, Chondrex, cat. 7024) at 2 mg/ml or with incomplete Freund’s adjuvant (IFA, Chondrex, cat. 7002) on day 0 and day 21. Mice were randomly assigned to treatment groups upon the establishment of arthritis. The number of animals used in each experiment is described in the corresponding schematic (Fig 1). For proteomics analysis, serum was collected at one week after intravenously injection of hUCB-MSCs or CS10 (100 μL). Serum was collected after centrifugation at 3,000 g for 15 min at room temperature. Clinical symptoms of arthritis were then monitored. hUCB-MSCs or CS10 were intravenously injected three times for two weeks. Clinical symptoms of arthritis were monitored three times per week from day 22 by two independent observers. Clinical arthritis indices (CAIs) of peripheral joints were quantified using a graded scale from 0 to 4 at as previously described [23]. Mean clinical score was determined on a graded scale from 0 to 4 as previously described.

### LC-MS/MS analysis

We performed a label-free quantitative proteomic analysis for CIA mice serum samples using an LC-MS/MS system (E-biogen, Inc., Korea) according to the manufacturer’s instructions. Briefly, we conducted proteomic analysis for serum samples (100 ug) (each group, n = 3) using a UPLC Exactive equipment. LC-MS/MS data were analyzed using MaxQuant and Perseus. Uniprot’s mouse database and LFQ (Label Free Quantification) were also used.

### Cell culture and secretome analysis

The hUCB-MSCs from the umbilical cord blood of healthy donors were isolated, expanded, and packaged according to the internal protocol at the GMP facility of Kangstem Biotech Co., Ltd, South Korea. The hUCB-MSCs were maintained in KSB-3 complete medium (Kangstem Biotech, South Korea) supplemented with 10% fetal bovine serum (FBS; Gibco, USA). The cells were incubated at 37°C in a CO2 incubator. All experiments described below were conducted by using hUCB-MSCs in passage 5. The information about the sex of the donors and culture doubling time are shown in S1 Table in S1 File. The differentiation potential of hUCB-MSCs and MSC surface marker profiles were examined. In the FACS analysis, hUCB-MSCs were positive for typical MSC markers CD44, CD73, and CD105, and negative for cell lineage markers CD11b, CD34, CD45, HLA-DR, and CD19 (S2 Table in S1 File). The percentage of positive staining was indicated to be more than 95% and negative staining was indicated to be less than 5% (S3 Table in S1 File). Tri-lineage differentiation was experimented with using a StemPro differentiation kit (Gibco, USA). The hUCB-MSC was seeded at 3x10^5 in 6 well plates and cultured for 3 weeks with a StemPro osteogenesis differentiation kit. Osteogenic differentiation was assessed by Alizarin Red staining. The hUCB-MSC was seeded at 2x10^5 in 6 well plates and cultured for 3 weeks with a StemPro Adipogenesis differentiation kit. Adipogenic differentiation was assessed by Oil Red O staining. The hUCB-MSC was seeded at 3x10^5 in a 15mL tube and cultured for 3 weeks with a StemPro chondrogenesis differentiation kit. Osteogenic differentiation was assessed by Alcian blue staining (S1 Fig).

For secreted protein analysis, cell culture supernatants were collected from three lots of hUCB-MSCs that were isolated from three donors. Cells (1 × 10^6^/well) were cultured in a 6-well plate with 2 mL KSB-3 Basal medium for 48 h. Secretome analysis of culture media was performed using Human Antibody Array L-1000 (Cat# AAH-BLG-1000-4; RayBiotech, USA) according to the manufacturer’s instructions by E-biogen, Inc. (Korea). Slide scanning was performed using a GenePix 4100A Scanner (Axon Instrument, USA). After scan images were obtained, they were grided and quantified with a GenePix Software (Axon Instrument, USA). Data about protein information were annotated using UniProt DB.

### DATA analysis

Data are presented with ExDEGA v1.2.1.0 software (EBIOGEN, Inc., Seoul, Korea). Data’s standard fold change was 1.5 at *P* < 0.05 in pathway enrichment analysis. Scatter plot in ExDEGA’s standard fold change was 1.5 at *p* < 0.05. Gene Ontology (GO) is an annotation technique that concentrates on genes based on high-throughput genomic or transcriptome data. GO analysis includes three independent categories. Of them, biological processes (BP) were analyzed. GO was performed using DAVID (The Database for Annotation, Visualization and Integrated Discovery, https://david.ncifcrf.gov/). The cut-off point was set at gene count > 2 and *p* < 0.05. We also performed hierarchical clustering (HCL) analysis using MeV software.

### Statistical analysis

All data were analyzed using GraphPad Prism version 5 (GraphPad Software) and expressed as mean ± standard deviation (SD). Clinical severity and Incidence rates of the CIA mice model were analyzed by Kruskal-Wallis test with Dunn’s multiple comparisons test. Statistical comparisons were performed using GraphPad Prism version 5 (T-test) and *p* < 0.05 was considered statistically significant.

## Results

### Administration of hUCB-MSCs shows therapeutic effects on the CIA mice model

In order to investigate the therapeutic efficacy and action mechanism of hUCB-MSC in RA, our study was conducted with a CIA mice model induced by immunizing mice with collagen type II (CII) in CFA or IFA. The overall experimental scheme is shown in Fig 1.

After the second immunization, two different doses (2 × 10^6^ cells, 3 × 10^6^ cells) of hUCB-MSCs were repeatedly administered by intravenous injection (IV) to CIA mice model three times per week. Clinical assessments including clinical arthritis index (CAI) scoring, paw volume, and body weight were performed daily for 44 days as the observation period. RA progression is known to be the most active. This time is also an appropriate time to observe pathological factors regulated by hUCB-MSCs [24]. Thus, mice sera were prepared at one week after the first injection of hUCB-MSCs. LC-MS/MS analysis was then performed. To investigate the correlation between candidates expected to be regulated by hUCB-MSCs identified by LC–MS/MS and their putative regulators secreted by hUCB-MSCs, antibody-based cytokine array was carried out using conditioned medium (CM) obtained by culturing hUCB-MSCs.

**Fig 2:**
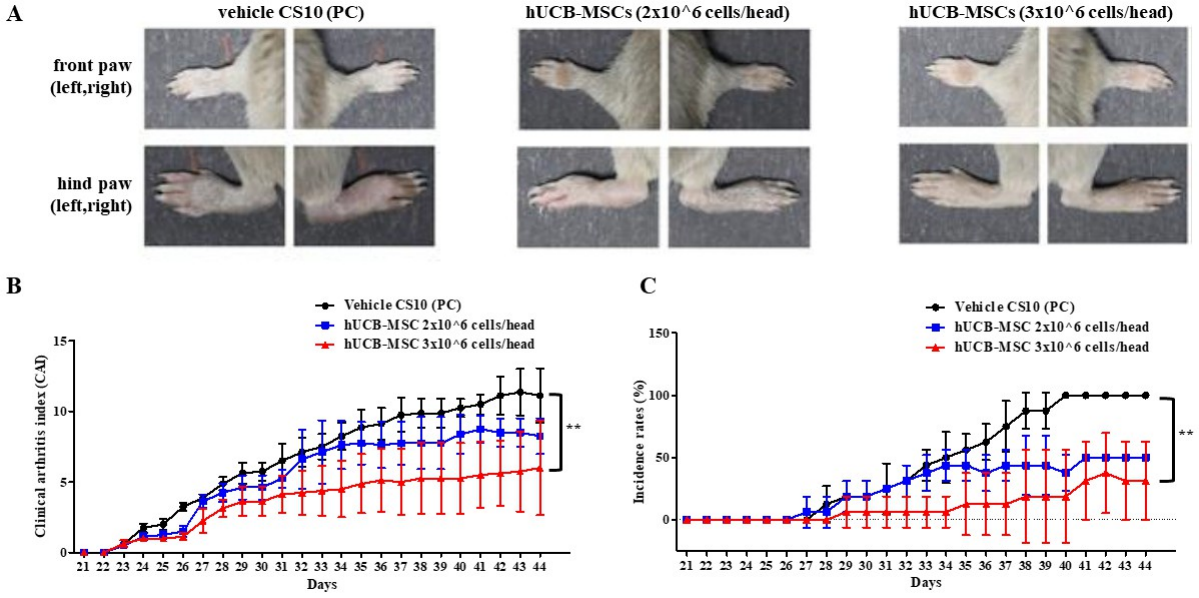
Effects of repeated injection of hUCB-MSCs in type II CIA model. (A) Representative images show gross features of front and hind paws at 44 days after immunization for all three animal groups. (B) Clinical scores of arthritic symptoms assessed by a visual arthritis scoring system were plotted daily until 44 days (n = 4 for each group). (C) Incidence rates of vehicle CS10 and hUCB-MSCs treated mice were plotted daily until 44 days (n = 4). Each point on the graph is presented as mean ± SD. Normality tests were performed using the Shapiro-Wilk normality test. Statistical significance versus vehicle CS10-treated CIA control group (as a control group) was determined by Kruskal-Wallis test with Dunn’s multiple comparisons test (** p < 0.01).

For vehicle (cryostor CS10) injected CIA mice in the PC group, their paws showed inflammatory signs of arthritis such as erythema and edema. After 44 days, paws of all mice in the PC group showed signs of arthritis. On the contrary, in the group administered with hUCB-MSCs, RA symptoms of CIA mice model were gradually reduced. Although the results did not show statistical significance between the doses of hUCB-MSCs, the therapeutic efficacy seemed to be significantly prominent at a higher dose. This trend was observed commonly for both CAI score and incidence rate (Fig 2B and 2C).

### Characterizing the proteomic profile of rheumatoid arthritis using sera of CIA mice by LC-MS/MS

**Fig 3:**
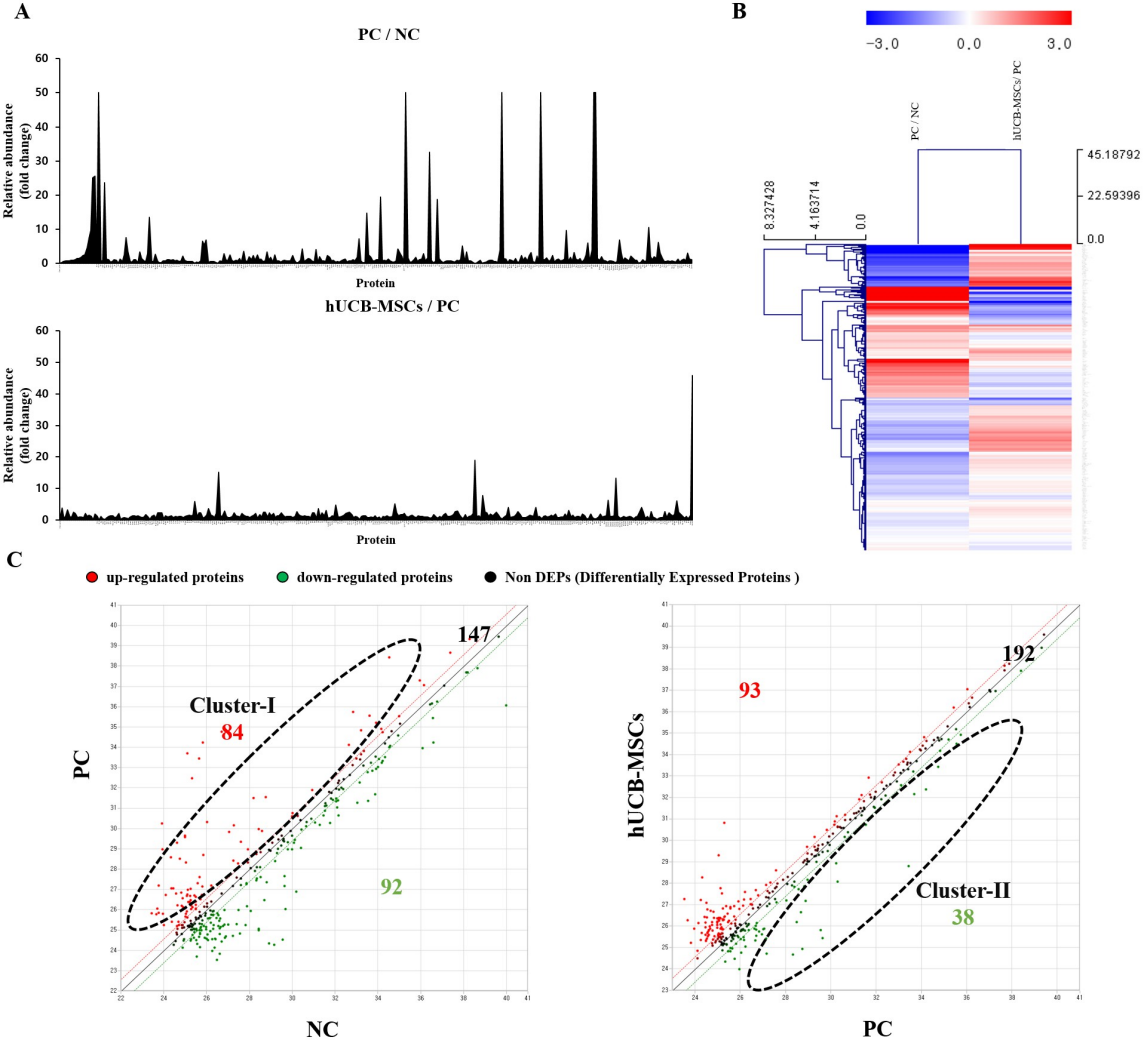
LC–MS/MS based serum proteomics for identification of candidate biomarkers for RA. (A) A graph comparing normalized values of genes analyzed by LC–MS/MS with the PC group and hUCB-MSCs/PC group. The x-axis shows the peak based on the protein and the y-axis shows the fold change value. (B) Heatmap analysis of genes of the PC group and hUCB-MSCs/PC group analyzed by LC–MS/MS using MEV program. Columns represent abundance with color as indicated in the legend (blue, lower abundance; red, higher abundance). (C) A scatter plot comparing gene expression levels between NC & PC and PC & hUCB-MSCs. The cluster-I shows up-regulated proteins in the PC group compared to the NC group. Cluster-II shown down-regulated proteins of hUCB-MSCs group compared to the PC group. The numbering standard is 1.5 or more based on the fold threshold. Red dots are up-regulated proteins. Green dots are down regulated proteins and black dots are proteins with a fold change of 1.5 or less.

To elucidate the underlying molecular mechanisms based on RA progression and hUCB-MSCs administration, LC-MS/MS analysis was conducted for serum samples of vehicle CS10 group (PC, RA-induced vehicle control group) and hUCB-MSCs group, and the results were compared with NC group (RA non-induced group). In addition, to investigate pathophysiologic factors regulated by hUCB-MSCs treatment, hUCB-MSCs administered mice group was compared to the PC group (Fig 3A). Heat map analysis was performed to confirm the overall pattern of proteins whose expression levels were changed during RA progression or changed after administration of hUCB-MSCs (Fig 3B). A total of 176 differentially expressed proteins (DEPs) were detected from PC group compared to NC group, including 84 up-regulated (Cluster-I) and 92 down-regulated ones. A total of 131 DEPs were detected from hUCB-MSCs group compared to PC group, including 93 up-regulated (Cluster-II) and 38 down-regulated ones. To characterize DEPs in functional groups and identify pathways significantly regulated by hUCB-MSCs in RA, we performed DAVID GO analysis.

The most abundant GO terms of Cluster-I were mainly acute-phase response (GO:0006953), innate immune response (GO:0045087), immune system process (GO:0002376), positive regulation of B cell activation (GO:0050871), and antigen processing and presentation (GO:0019882). These results suggest that while the CIA mice model has remained different features from human clinical symptoms, it still exists limitations as an experimental model to study RA. The most abundantly up-regulated proteins and prominent properties in the PC group were associated with RA humoral autoimmune process. GO terms related to platelet activation, such as complement activation (GO:0006958) and blood coagulation (GO:0007596) were also noticed (Table 1).

**Table 1:** Gene Ontology (GO) analysis of PC group and hUCB-MSCs group by LC-MS/MS.

(A) The biological processes with up-regulated gene enrichment scores in PC group			
**GO ID**	**Functional Category**	**Genes**	**P-Value**
GO:0006953	acute-phase response	CRP, ORM1, SAA1, FN1, HP, SAA2, LBP, SERPINA3N, ORM2	4.E-13
GO:0045087	innate immune response	C3, IGHM, APCS, IGHG1, CFH, SLPI, LCN2, PTX3, LBP, IGLC2, CD14, S100A9	2.E-07
GO:0002376	immune system process	C3, CFH, SLPI, CD5L, LCN2, HP, LBP, CD14, S100A9, PSMB8, LTF	1.E-06
GO:0006508	Proteolysis	PM20D1, F7, PSMB2, PSMA2, CTRB1, DPEP2, PROZ, HP, CTSS, PSMB8, MMP10, LTF	8.E-06
GO:0006958	complement activation, classical pathway	CRP, C3, IGHM, IGHG1, IGLC2	5.E-05
GO:0007596	blood coagulation	C3, F7, PROZ, HRG	4.E-03
GO:0050871	positive regulation of B cell activation	IGHM, IGHG1, IGLC2	6.E-03
GO:0006879	cellular iron ion homeostasis	HPX, TFRC, LCN2	1.E-02
GO:0019882	antigen processing and presentation	IGHM, CTSS, PSMB8	2.E-02
GO:0030194	positive regulation of blood coagulation	F7, S100A9	5.E-02
(B) The biological processes with down-regulated gene enrichment scores in hUCB-MSCs/PC group			
**GO ID**	**Functional Category**	**Genes**	**P-Value**
GO:0006953	acute-phase response	ORM1, SAA2, ORM2	4.E-04
GO:0006096	glycolytic process	ALDOB, BPGM, ALDOA	5.E-04
GO:0070527	platelet aggregation	PDGFRA, TLN1, VCL	5.E-04
GO:0048525	negative regulation of viral process	APCS, LTF	4.E-03
GO:0061615	glycolytic process through fructose-6-phosphate	ALDOB, ALDOA	4.E-03
GO:0002682	regulation of immune system process	ORM1, ORM2	5.E-03
GO:0006000	fructose metabolic process	ALDOB, ALDOA	8.E-03
GO:0030388	fructose 1,6-bisphosphate metabolic process	ALDOB, ALDOA	8.E-03

Results of GO analysis indicated that most DEPs in Cluster-II were simultaneously enriched in acute-phase response (GO:0006953) and regulation of immune system process (GO:0002682). In addition, certain DEPs in Cluster-II were closely associated with regulation of platelet activation such as platelet aggregation (GO:0070527) (Table 1). These data suggest that hUCB-MSCs administration has the potential to modulate acute phase protein biomarker level, various protein involved in humoral immunity, and platelet activation.

### Interpreting results of hUCB-MSCs analysis with L-1000

**Fig 4:**
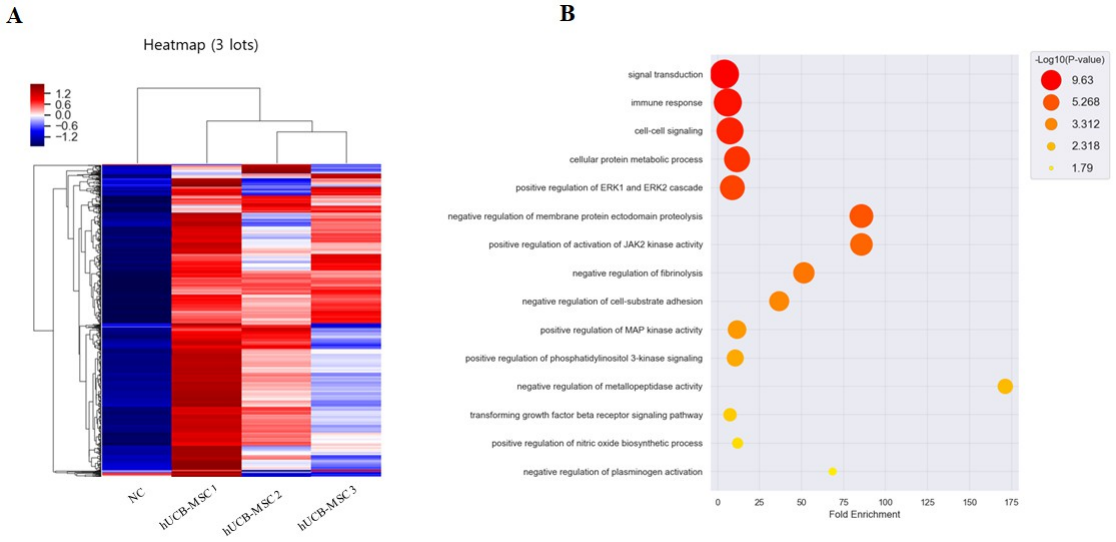
L-1000 analysis of cell culture supernatants of hUCB-MSCs for identification of secreted protein about regulating RA. (A) Heatmap analysis of genes in three samples after treatment with hUCB-MSCs analyzed by L-1000 using MEV program. Colors of columns represent relative abundances. Columns indicate the abundance with different colors as indicated in the legend (blue, lower abundance; red, higher abundance). (B) GO terms in the enrichment analysis of genes affected by hUCB-MSCs. Higher significance is indicated by red and lower significance is indicated by yellow as described in the legend. The position of the circle changes according to fold enrichment.

To explore the factors involved in the enhancement of therapeutic efficacy by hUCB-MSCs, the cytokine profile of the conditioned media (CM) was analyzed using an antibody-based cytokine array. MSC populations derived from three different donors were prepared. Their cytokine secretion profiles were found to exhibit donor-dependent different patterns (Fig 4A). Of a total of 1,000 antibody probes, 100 probes showed 1.5-fold or more expression compared to the NC group in 3 CM in common. GO annotation was performed for these 100 candidates (Fig 4B). As shown in Table 2, GO terms were mainly associated with signal transduction (GO:0007165), immune response (GO:0006955), cell-cell signaling (GO:0007267), and cellular protein metabolic process (GO:0044267). They also included diverse signaling pathways such as ERK1/2, JAK kinase, MAP kinase, and phosphatidylinositol (PI)-3 kinase signaling. Interestingly, three donor’s hUCB-MSCs also commonly secreted effective factors belonging to GO terms involved in regulating agonist of platelet activation such as negative regulation of fibrinolysis (GO:0051918) and negative regulation of plasminogen activation (GO:0010757).

**Table 2:** Gene Ontology (GO) analysis of hUCB-MSCs by L-1000.

GO ID	Functional Category	Genes	P-Value
GO:0007165	signal transduction	GRN, SPARC, TRADD, CXCL1, WISP1, LGALS1, PLAU, MDK, WIF1, TNFSF10, CD38, IGFBP1, FGA, SMAD1, TNFSF12, VEGFC, IL19, IL17RD, LYVE1, SMAD5, TLR1, CXCL11, S100A6, TNFSF8, TEK, ROR2, IL7R, MET	2.E-10
GO:0006955	immune response	CCL25, EDA, TNFSF12, GZMA, IL19, CXCL1, THBS1, TLR1, VTN, CXCL11, TNFSF10, TNFSF8, PGLYRP1, IL7R, B2M	1.E-07
GO:0007267	cell-cell signaling	PYY, CXCL11, TNFSF10, TNFSF8, TEK, INHBA, GDF5, PTHLH, AGT, WISP1, INS	2.E-06
GO:0044267	cellular protein metabolic process	IGFBP1, FGA, APP, MMP1, PLG, TGFBI, B2M, INS	5.E-06
GO:0070374	positive regulation of ERK1 and ERK2 cascade	CCL25, FGA, BMPER, PDGFC, VEGFB, TEK, MIF, FGF23, VEGFA	8.E-06
GO:0051045	negative regulation of membrane protein ectodomain proteolysis	TIMP2, TIMP3, TIMP1	5.E-04
GO:0010535	positive regulation of activation of JAK2 kinase activity	IL23A, IL23R, AGT	5.E-04
GO:0051918	negative regulation of fibrinolysis	SERPINE1, PLG, THBS1	1.E-03
GO:0010812	negative regulation of cell-substrate adhesion	LGALS1, FZD4, PLG	3.E-03
GO:0043406	positive regulation of MAP kinase activity	PDGFC, KRAS, MIF, VEGFA	5.E-03
GO:0014068	positive regulation of phosphatidylinositol 3-kinase signaling	PDGFC, TEK, AGT, INS	6.E-03
GO:1905049	negative regulation of metallopeptidase activity	TIMP2, TIMP1	1.E-02
GO:0007179	transforming growth factor beta receptor signaling pathway	SMAD1, SMAD4, GDF5, SMAD5	2.E-02
GO:0045429	positive regulation of nitric oxide biosynthetic process	CLU, AGT, INS	3.E-02
GO:0010757	negative regulation of plasminogen activation	SERPINE1, THBS1	3.E-02

## Discussion

Therapeutic effects of MSCs on diverse autoimmune diseases, including multiple sclerosis (MS), systemic lupus erythematosus (SLE), autoimmune hepatitis (AIH), atopic dermatitis (AD), type 1 diabetes, Crohn`s disease, and RA, have been investigated [25, 26]. In particular, the use of MSCs has been considered as a promising alternative therapeutic tool in RA because a significant number of RA patients could not tolerate current anti-rheumatic drugs [27]. While numerous studies have been conducted to evaluate the effectiveness of MSCs on RA in animal studies and RA patient’s clinical trials, they have demonstrated inconsistent results. A previous meta-analysis of 30 studies in which MSCs were administered to an RA animal model has revealed that such discrepancy results might be due to factors such as the source of MSCs, administration timing, route of administration, MSC dose, and treatment regimen [28].

Regarding mechanisms involved in therapeutic effects of MSCs on RA, it is known that MSCs possess immunomodulatory functions on diverse immune cells. MSCs can inhibit the proliferation or activation of various inflammatory immune cells such as T cells, B cells, monocytes, macrophage, natural killer cells, and dendritic cells [29, 30]. MSCs may slant the Treg/Th17 balance toward Treg cells and modulate the transition of Th1 to Th2 cells [31, 32]. MSCs may also facilitate M1 to M2 macrophage transition [33, 34]. During restoration of immune balance by MSCs, levels of various pro-inflammatory cytokines such as TNF-α, IL-1, IL-6, and IL-17 associated with RA pathology are decreased while levels of anti-inflammatory cytokine IL-10 are increased [35–37]. Consistent with these reports, our previous study has also shown that when hUCB-MSCs are administered to CIA mice model, levels of TNF-α, IL-1, and IL-6 are decreased while levels of IL-10 are increased at inflamed tissues. The population of CD4+CD25+FOXP3+ Treg cells is also increased. Most of these changes were induced by MSCs in a dose-dependent manner [38]. However, since most studies have targeted specific cell types or specific cytokines, there are still many questions about the mechanism of action underlying MSC therapy in RA animal model. To better understand RA pathogenesis as well as the mechanism of action underlying MSC therapy, a comprehensive analytic method is needed.

In this study, we performed quantitative profiling for hundreds of known metabolites using CIA mice serum samples by LC-MS/MS analysis, a more comprehensive and sensitive tool. After inducing CIA in mice through two immunizations according to a conventional method, hUCB-MSCs were administered intravenously at two doses (2 × 10^6^ cells; 3 × 10^6^ cells) three times. Compared to the PC group, the arthritis severity and incidence rate of inflamed paw were significantly suppressed in both hUCB-MSCs groups in proportion to the dose of MSCs injected. Using mice serum samples, we performed LC–MS/MS analysis. To elucidate mechanisms of RA progression and hUCB-MSCs efficacy, differently expressed cytokines were grouped and functionally categorized based on GO annotation of biological process. It is generally known that RA is a specific humoral or cellular autoimmune disease to autoantigens. Pro-inflammatory cytokines induced by various immune reactions circulate in the body of RA patients and contribute to immune pathogenesis of this disease [39–41]. In this regard, the most enriched GO terms of up-regulated DEPs in CIA mice sera included innate immune response (GO:0045087), immune system process (GO:0002376), and antigen processing and presentation (GO:0019882). In RA clinical patients, concentrations of several acute-phase proteins can provide useful diagnostic information. The most frequently used acute-phase proteins as RA biomarkers are erythrocyte sedimentation rate (ESR), C-reactive protein (CRP), serum amyloid A protein (SAA), alpha-1-acid glycoprotein (AGP or ORM), haptoglobin (HP), and alpha-1-antitrypsin (AAT) [42, 43]. These expression levels are usually correlated with clinical assessment of disease severity. In our CIA mice model, various acute-phase proteins (GO:0006953) were also significantly elevated, including CRP, SAA, ORM, and HP, similar to results found in human RA patients. Interestingly, among these RA biomarkers, ORM and SAA were down-regulated along with a decrease of CAI score in the of hUCB-MSCs group as a result of therapeutic efficacy.

Although platelets are mainly known for their hemostatic functions, they also play a substantial role in RA pathophysiology. In RA patients, platelet agonists such as ADP, collagen, fibrinogen, plasmin, and pro-inflammatory cytokines are dysregulated, resulting in platelet abnormal activation. The cascade of activated platelet signaling can promote the secretion of RA inducers or formation of MPs [44, 45]. It may further instigate rapid communications between platelets and various immune cells. Platelets react on almost all immune cell types. B cell and T cell activation might be induced by their soluble and membrane bound CD40 ligand. By direct cell to cell interaction with Th1, Th17 or Treg cell, platelets can modulate these cells`differentiation or secretion level of pro-inflammatory cytokines. MPs of platelets and their cargo are internalized by neutrophils to trigger neutrophil degranulation [46–48]. Although platelets are known to react with such a variety of immune cells related to RA pathology, the interaction between platelets and MSCs has not been fully elucidated yet. In our CIA mice model, complement activation (GO: 0006958) and blood coagulation (GO: 0007596) related to platelet activation GO term were also enriched in CIA mice sera. However, platelet aggregation (GO:0070527) was negatively regulated in sera samples of hUCB-MSCs administered mice. To investigate the evidence of this platelet regulation by hUCB-MSCs, we prepared CM samples from three different donors and performed L-1000 human antibody-based cytokine array using these CM samples. This L-1000 array consisted of antibodies against 1000 cytokines, and the result of secretome analysis demonstrated donor-specific features. Among a total of 1,000 antibody probes, 100 probes were commonly detected to be upregulated for more than 1.5-fold in three CM samples compared to those in NC sample (blank media). When we performed GO annotation for those 100 proteins, typical characteristics of MSC secretion factors such as signal transduction (GO:0007165), immune response (GO:0006955), and cell-cell signaling (GO:0007267) were ranked the highest. Interestingly, negative regulation of fibrinolysis (GO:0051918) and negative regulation of plasminogen activation (GO:0010757) GO terms were also noticed. Based on these annotation results, hUCB-MSC might be interpreted as having the capability to suppress the conversion of plasminogen to plasmin. In other words, hUCB-MSC might be able to inhibit production of platelet agonist. Therefore, the activity of platelet, which plays a major role in RA pathology, might be inhibited from the initial status.

According to a recent study, MSCs could inhibit platelet activation. This function is dependent on the expression level of CD73. The ectonucleotidase activity of this protein could convert AMP to adenosine. Subsequent activation of P1 receptor of platelet can increase its cytoplasmic cAMP and VASP phosphorylation levels. Finally, through a series of processes, MSCs could suppress excessive platelet activation [49]. Based on the above research, several research groups are in agreement that regulation of platelet activation will be included in the MSC treatment efficacy or immunomodulatory function in other disease models [50–52]. In our study, inhibiting plasmin production might inhibit platelet activation. Two possible candidate proteins, SERPINE1 and THBS1, might have such function. SERPINE1, better known by its previous symbol PAI- 1 (plasminogen activator inhibitor -1), belongs to a family of serine protease inhibitors. It is a key molecule for inhibiting urokinase (uPA) and tissue-type (tPA) plasminogen activators. In turn, SERPINE1 can inhibit their ability to convert plasminogen to plasmin [53]. THBS1 also has the potential to reduce plasmin level. While some studies have shown that THBS1 can enhance thrombin-stimulated platelet aggregation, other studies have reported that some monoclonal antibodies recognizing THBS1 can inhibit thrombin-stimulated platelet aggregation [54, 55]. Therefore, further studies on the precise mechanism of THBS1 involved in the regulation of platelet aggregation related to RA pathology are needed.

Taken together, besides their well-known immunomodulatory functions, hUCB-MSCs might have a novel therapeutic mechanism by inhibiting platelet activation based on the results of the present study. However, considering the conditions of the serum analysis, these results were limited to the early stage of RA progression. In addition, the main immunomodulatory factors of hUCB-MSCs were also limited to the common factors which were expressed in three lots under basic culture conditions. By expanding research on the action mechanism of MSCs and providing more scientific evidence, this study is expected to build a more effective therapeutic and application protocol for using hUCB-MSC in RA.

## Conclusion

In summary, we investigated the mechanism of RA progression and effects of hUCB-MSCs treatment in a more comprehensive manner by cross-analyzing sera of RA mice model using LC–MS/MS analysis and antibody-based cytokine array of hUCB-MSCs. Results of this study suggest that platelet regulation might be a novel target of RA, which is expected to be regulated by hUCB-MSCs. Our analysis provides a new perspective on the treatment of RA, which will help advance our understanding of hUCB-MSCs and their therapeutic mechanisms in RA.

## Supporting information

S1 Fig Differentiation potential of hUCB-MSCs. hUCB-MSCs have a potential for Tri-lineage differentiation into osteoblasts (Alizarin Red), adipocytes (Oil Red O staining) and chondroblasts (Alcian Blue). (original magnification in 1), ×40; in 2) and 3) ×200).
(TIF)

S1 File (DOCX)
