# Supplementary figures and images for: LC-MS/MS-based serum proteomics reveals a distinctive signature in a rheumatoid arthritis mouse model after treatment with mesenchymal stem cells
Source: PLoS One. 2022 Nov 4;17(11):e0277218. doi: 10.1371/journal.pone.0277218 (PMC9635733; doi:10.1371/journal.pone.0277218)

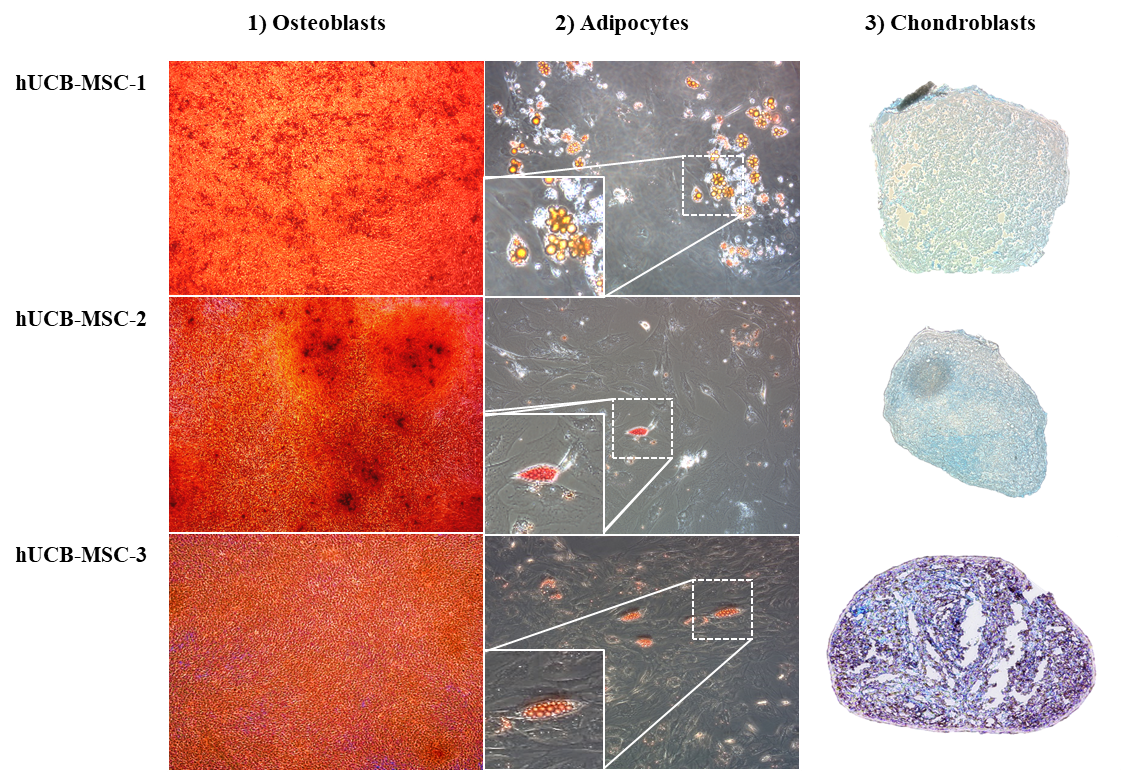

Supplement: S1 Fig — hUCB-MSCs have a potential for Tri-lineage differentiation into osteoblasts (Alizarin Red), adipocytes (Oil Red O staining) and chondroblasts (Alcian Blue). (original magnification in 1), ×40; in 2) and 3) ×200). (TIF) [file pone.0277218.s001.tif]
