# Supplementary material for: LC-MS/MS-based serum proteomics reveals a distinctive signature in a rheumatoid arthritis mouse model after treatment with mesenchymal stem cells
Source: PLoS One. 2022 Nov 4;17(11):e0277218. doi: 10.1371/journal.pone.0277218 (PMC9635733; doi:10.1371/journal.pone.0277218)
Supplement: S1 File — (DOCX) [file pone.0277218.s002.docx]

**Supporting Information**

**S1 Table** The sex of the donors and culture doubling time

|  | The sex of the donors | Doubling time (hours) | | | |
| --- | --- | --- | --- | --- | --- |
|  |  | Passage 1 | Passage 2 | Passage 3 | Passage 4 |
| hUCB-MSC-1 | Male | 16.31 | 20.29 | 21.61 | 23.24 |
| hUCB-MSC-2 | Female | 15.6 | 19.1 | 22.8 | 39.8 |
| hUCB-MSC-3 | Female | 18.5 | 20.3 | 30.6 | 41.8 |

**S2 Table** Antibodies used for flow cytometry

|  | **Product name** | **isotype** | **Manufacturer** | **Cat no.** |
| --- | --- | --- | --- | --- |
| **Control** | FITC Mouse IgG1, κ Isotype Control | Mouse Control IgG1 | BD Bioscience | 555748 |
| **Positive Marker** | FITC Mouse Anti-Human CD44 | Mouse BALB/c IgG1, κ |  | 347943 |
|  | PE Mouse Anti-Human CD73 | Mouse IgG1, κ |  | 550257 |
|  | PE Mouse anti-Human CD105 | Mouse BALB/c IgG1, κ |  | 560839 |
| **Negative Marker** | PE Mouse Anti-Human CD11b | Mouse IgG1, κ |  | 555388 |
|  | FITC Mouse Anti-Human CD34 | Mouse IgG1, κ |  | 555821 |
|  | FITC Mouse Anti-Human CD45 | Mouse IgG1, κ |  | 555482 |
|  | FITC Mouse Anti-Human CD19 | Mouse IgG1, κ |  | 555412 |
|  | FITC Mouse Anti-Human HLA-DR | Mouse IgG2b, κ |  | 555560 |

**S3 Table** Expression of MSC surface makers of hUCB-MSCs

| Marker  No. | **Positive Markers (%)** | | | | **Negative Markers (%)** | | | |
| --- | --- | --- | --- | --- | --- | --- | --- | --- |
|  | CD44 | CD73 | CD105 | CD11b | CD34 | CD45 | HLA-DR | CD19 |
| hUCB-MSC-1 | 99.19 | 99.4 | 98.26 | 3.15 | 0.65 | 0.98 | 0.76 | 3.18 |
| hUCB-MSC-2 | 97.28 | 99.16 | 98.3 | 3.36 | 1.61 | 1.93 | 2.26 | 3.8 |
| hUCB-MSC-3 | 96.03 | 99.52 | 98.49 | 0.17 | 0.06 | 0.14 | 0.03 | 0.16 |

hUCB-MSCs were positive for typical MSC markers CD44, CD73 and CD105, and negative for cell lineage markers CD11b, CD34, CD45,HLA-DR and CD19. The percentage of the positive staining was indicated more than 95% and negative staining was indicated less than 5%.
